# Cell Intrinsic and Systemic Metabolism in Tumor Immunity and Immunotherapy

**DOI:** 10.3390/cancers12040852

**Published:** 2020-04-01

**Authors:** Michael F. Coleman, Alyssa J. Cozzo, Alexander J. Pfeil, Suhas K. Etigunta, Stephen D. Hursting

**Affiliations:** 1 Department of Nutrition, University of North Carolina, Chapel Hill, NC 27516, USA; mcoleman@unc.edu (M.F.C.); ajc1983@live.unc.edu (A.J.C.); pfeilal@live.unc.edu (A.J.P.); suhase@live.unc.edu (S.K.E.); 2 Department of Medicine, Duke University, Durham, NC 27705, USA; 3 Lineberger Comprehensive Cancer Center, University of North Carolina, Chapel Hill, NC 27516, USA

**Keywords:** immune checkpoint inhibition, metabolism, cancer, obesity, calorie restriction

## Abstract

Immune checkpoint inhibitor (ICI) therapy has shown extraordinary promise at treating cancers otherwise resistant to treatment. However, for ICI therapy to be effective, it must overcome the metabolic limitations of the tumor microenvironment. Tumor metabolism has long been understood to be highly dysregulated, with potent immunosuppressive effects. Moreover, T cell activation and longevity within the tumor microenvironment are intimately tied to T cell metabolism and are required for the long-term efficacy of ICI therapy. We discuss in this review the intersection of metabolic competition in the tumor microenvironment, T cell activation and metabolism, the roles of tumor cell metabolism in immune evasion, and the impact of host metabolism in determining immune surveillance and ICI therapy outcomes. We also discussed the effects of obesity and calorie restriction—two important systemic metabolic perturbations that impact intrinsic metabolic pathways in T cells as well as cancer cells.

## 1. Introduction

Cancer immunotherapies are paradigm-shifting drugs employed to augment the host antitumor immune response to treatment. The subset of immunotherapies known as immune checkpoint inhibitors (ICI) promote effective antitumor immunity by relieving suppressive effects induced by various immune checkpoint signaling pathways in T cells. Immune checkpoints refer to numerous inhibitory pathways that are necessary for self-tolerance and modulation of the immune response [1,2]. Antibodies targeting cytotoxic T lymphocyte antigen-4 (CTLA-4) (ipilimumab and tremelimumab), programmed death-1 (PD1) (nivolumab and pembrolizumab), and programmed death lignd 1 (PD-L1) (atezolizumab, avelumab, and durvalumab) are currently approved ICI therapies that have yielded dramatic successes in the treatment of blood cancers, such as multiple myeloma [1]. Some solid tumors have shown poor response to ICIs (e.g., pancreatic cancers), while, in others (e.g., melanoma and lung cancer), these drugs promote effective antitumor immunity in a modest subset of treated patients, resulting in a substantial extension of progression-free survival [2]. However, a larger subset of patients sees limited benefit from ICI treatment [2]. Thus, determining the mechanisms underlying nonresponse or resistance following initial response to ICI therapy will be critical in extending the utility of these drugs. At present, ICI therapy is limited by the lack of widely applicable screening approaches to identify responders and nonresponders pretreatment [2]. Moreover, the high financial cost and potential for serious adverse events pose additional challenges to safe and effective treatment of patients with ICIs [1].

In recent years, immunotherapy and immunometabolism have advanced rapidly. Arising from such rapid evolution is a series of knowledge gaps, particularly in the area of immunometabolic determinants of immunotherapy outcomes. The tumor microenvironment (TME) comprises a multitude of cell types in various metabolic states, whose uptake and export of metabolites determine the availability of each metabolite to other cells in the TME [3]. This cellular complexity results in a profile of nutrient availability that is starkly different from that of the corresponding normal tissue [4]. The TME is metabolically hostile to immune cells, in part due to the existence of significant competition between various cell types for a limiting nutrient pool [5]. Such aberrant metabolite abundance is further compounded by inefficient tumor vasculature, resulting in frequent zones of hypoxia and differential metabolism [6]. Here, we considered the role of microenvironment nutrient profiles in shaping tumor immune surveillance and the potential for crosstalk between immunotherapy, cell metabolism, and energy balance.

To execute their function, immune cells undergo rapid, phenotypic shifts in response to stimuli in their environment. Such phenotypic shifts are often supported by radical reprogramming of immune cell metabolism, failure of which results in impotent immune cell response, ultimately permissive of the instigating stimulus [7]. In the case of cancer, metabolic suppression of T cell activation is a critical impediment to antitumor immunity [8,9]. Given that effective immunotherapy requires rapid metabolic reprogramming of T cells, immunotherapy is, in part, a metabolic therapy. Thus, understanding TME metabolic interactions will be essential to advances in ICI therapies.

Understanding how metabolism shapes immunotherapy response, and ultimately resistance, will inform novel strategies to increase both the tumor types targeted by immunotherapy and the rate of immunotherapy response. Advances in our understanding in these areas may allow for the generation of metabolic biomarkers of immunotherapy response. As have been discussed herein, much work is already underway to determine synergistic combinations of metabolic interventions with ICIs. This review aimed to examine the metabolic determinants of ICI response, both immune cell-intrinsic and extrinsic, including TME nutrient availability and metabolic mechanisms through which tumor cells evade immune surveillance.

## 2. Microenvironmental Metabolic Complexity

The TME is a dynamic intercellular communication network between malignant and nontransformed cells modulated by a complex mixture of metabolites, cytokines, and growth factors [10,11]. The heterogeneous cell populations of the TME compete for limited metabolic resources [12]. While it has been well documented that proliferating tumor cells upregulate glucose consumption, activated cytotoxic T cells also rely on aerobic glycolysis and enhanced glucose uptake [13,14]. Competition between tumor and cytotoxic T cells for limited glucose in the TME hampers immune response [5,15]. While cytotoxic CD8^+^ T cell function would be impaired in the low glucose conditions of the TME, T regulatory cells (Tregs), which rely on fatty acid oxidation (FAO) rather than glycolysis, can survive and exert their immunosuppressive effects [16]. Interestingly, ICI resistance arising from metabolic competition has been mitigated by enhanced fatty acid metabolism in cytotoxic T cells [17]. Similarly, the peroxisome proliferator-activated receptor α (PPARα) agonist bezafibrate has promoted response to anti-PD1 therapy by T cells via metabolic reprogramming [18,19,20].

Some innate immune cells also shift from oxidative phosphorylation (OXPHOS) to glycolysis upon activation [21]; for example, dendritic cells (DCs) require increased glucose consumption upon activation [22,23]. In contrast, tolerogenic DCs, associated with immunosuppression, display a metabolic signature of enhanced oxidative phosphorylation that regulates their tolerogenic function [24]. Metabolic competition for glucose between tumor cells and antigen-presenting cells (APCs), coupled with the subsequent shift from glycolysis to OXPHOS of APCs, could further explain the lack of T cell effector function and overall immunosuppressive role of the TME. Importantly, the limited efficacy of immunotherapies may be driven by reduced metabolic substrate availability for immune populations arising from metabolic competition in the TME.

## 3. Tumor Cells

Tumor cell metabolism is characterized by increased utilization of aerobic glycolysis, altered mitochondrial metabolism, and enhanced amino acid uptake and metabolism [25]. Tumor cell metabolism is further complicated by limited nutrient and oxygen abundance, successful adaptation to which is essential for tumor cell survival [26]. The metabolite profile of tumor interstitial fluid (TIF) is highly distinct from plasma and is modulated by the anatomical site of the tumor, tumor cell genetics, and diet [4]. TIF composition reflects an integral of the rate of nutrient supply (informed by dysfunctional vasculature and tissue site of origin), the rate of metabolite uptake by cells, and the rate of metabolite efflux from cells; thus, TIF composition reflects intra- and inter-tumoral heterogeneity [4]. Tumor cell metabolism is determined by various factors, including the balance between anabolic (often ATP-dependent) and ATP-producing reactions, maintenance of redox homeostasis, and adaptation to cellular/environmental stresses [26].

Mitochondrial dysfunction sits at a nexus of cancer cell metabolism and phenotypic plasticity [27]. Indeed, mitochondrial dysfunction has been shown to be critical to the development of invasive and metastatic phenotypes in some tumor models [28,29,30,31,32]. As such, mitochondrial dysfunction may offer competitive advantages for tumor cells. However, mitochondrial dysfunction can also result in limiting oxidized nicotinamide adenine dinucleotide (NAD^+^) levels, which remodels one-carbon metabolism [33] and cellular energy-sensing [34]. Loss of succinate dehydrogenase activity results in synthetic reliance on pyruvate carboxylase-derived oxaloacetate-mediated aspartate production [35], as well as remodeling of cellular metabolism through the stabilization of hypoxia-inducible factor 1α (HIF-1α) [36].

Tumor tissue of origin, as well as specific oncogene and tumor suppressor mutation combinations, contribute to significant metabolic heterogeneity in solid tumors. Activation of several oncogenes is associated with profound metabolic reprogramming of cancer cells towards maintaining anabolic metabolism despite the presence of various cellular stressors [37]. For example, KRAS (KRAS proto-oncogene, GTPase) mutation, common in lung, colorectal, and pancreatic cancers [38], supports tumor cell growth and survival in large part via remodeling of glucose metabolism, increasing non-oxidative pentose phosphate pathway flux to support nucleotide biosynthesis [39]. KRAS also drives glutamine metabolism to support redox homeostasis in cancer cells [40]. KRAS activation results in dramatic shifts in the metabolic fates of glucose and glutamine in the cell. Thus, not only is the flux through these pathways enhanced, but coordinated regulation is lost [41]. KRAS and MYC (MYC proto-oncogene, bHLH transcription factor) have been shown to cooperate in modulating cellular metabolism [42]. For this reason, delineating the relative contribution from MYC/KRAS-mediated dysregulation of metabolism is challenging [42]. Similarly, oncogenic activation of MYC promotes the upregulation of glycolysis while also promoting glutamine uptake and utilization [43]. PI3K/AKT activation downstream of these oncogenes promotes glucose uptake, glycolysis, and modulation of mitochondrial metabolism through both direct phosphorylation of metabolic proteins and the modulation of metabolic regulator cell signaling molecules [44,45].

The loss of tumor suppressors is a common feature in the development of cancer, typically occurring early and enabling the acquisition of further genetic lesions as tumors develop [25]. Several commonly mutated tumor suppressors are intimately interconnected with metabolism, and their mutational status can significantly inform tumor metabolic profile [46]. p53 is perhaps the most famous tumor suppressor, with important roles in development, aging, stem cell function, and diabetes in addition to its role in cancer [47]. p53 activation regulates glycolysis and OXPHOS, in part by repressing glucose transporters 1 and 4 (GLUT1/GLUT4) expression [48] and inducing TIGAR (TP53 (tumor protein P53)-inducible glycolysis and apoptosis regulator) [49]. p53 further modulates tumor metabolism via control of glutamine metabolism [50]. Thus, activation of p53 stunts fluxes through central carbon metabolism while promoting glutamine metabolism [51]. Loss of phosphatase and tensin homolog (PTEN) similarly regulates glucose metabolism through interaction with several key glycolytic proteins [52]. Further, PTEN loss enables enhanced pyrimidine biosynthesis and glutamine metabolism in cancer [53], while enhanced PTEN expression suppresses glutamine consumption to protect against cancer [54]. Loss of liver kinase B1 (LKB1) reduces oversight of cellular metabolism in part via suppression of adenosine monophosphate-activated protein kinase (AMPK) activation. AMPK activation promotes the reduction of biosynthetic metabolism crucial for rapid proliferation [55] and promotes OXPHOS via mitochondrial biogenesis [56]. Loss of kelch like ECH-associated protein 1 (KEAP1) results in the stabilization of nuclear factor erythroid 2 like 2 (NRF2) and increased cysteine demand, which has partially met the enhanced uptake of cysteine via antiport with nonessential amino acids [57]. This results in a synthetic dependence on nonessential amino acids to support cysteine metabolism in KEAP1 mutant lung cancer [57].

## 4. Fibroblasts

Cancer-associated fibroblasts (CAFs), a heterogeneous and plastic population, play important roles in tumor growth, survival, and metastasis. CAFs achieve this, in part, by supporting tumor cell metabolism and via the production of a range of growth factors, chemokines, and cytokines that promote immune suppression, angiogenesis, and tumor growth [58]. In addition, CAFs actively remodel the TME through lactate, amino acid, and lipid metabolism, generating nutrients required by tumor cells for metabolism [59]. CAFs may also directly promote immunosuppression via expression of PD-L1 and PD-L2 [60,61,62], promoting T cell anergy.

## 5. Macrophages

Tumor-associated macrophages (TAMs), thought to be derived from inflammatory monocytes [63], are critical to the development of an immunosuppressive TME [64] through the release of immunosuppressive cytokines, expression of immune checkpoint ligands, and the expression of nonclassical human leukocyte antigen (HLA) molecules, such as HLA-G and HLA-E. TAMs further maintain tumor immunosuppression by taking up and degrading anti-PD1 antibodies [65]. TAM function is heavily influenced by the surrounding microenvironment conditions. For example, hypoxia antagonizes mammalian target of rapamycin (mTOR) activation in TAMs and promotes FAO; this metabolic shift enables neighboring endothelial cells to facilitate metastasis [66]. High lactate levels within the TME have been shown to stabilize HIF-1α to support TAM/M2 like gene expression profiles [67]. TAMs also directly link microenvironment nutrient availability with immune function via the expression of arginase-1, which rapidly degrades arginine, thereby limiting T cell activation by stunting the arginine-dependent expression of T cell recptor ζ ( TCRζ) [68,69]. Arginine availability profoundly regulates T cell metabolism, survival, and antitumor activity [70]. TAMs express high levels of indoleamine dioxygenase (IDO), which has immunosuppressive effects on T cells [71,72] and cooperates with arginine metabolism to suppress APC function [73]. Thus, the relative abundance of TAMs and their metabolic plasticity plays a critical role in the establishment of an immunosuppressive TME and are a significant cause of immunotherapy resistance.

## 6. Dendritic Cells

DCs are APCs that bridge innate and adaptive immunity by activating CD8^+^ and CD4^+^ T cells via major histocompatibility complex class I or II (MHC-I or MHC-II), respectively [74]. In order to activate T cells, DCs must first themselves be activated by the recognition of damage-associated molecular patterns or pathogen-associated molecular patterns via highly conserved receptors, including nucleotide-binding oligomerization domain-like receptors (NOD-like-) and Toll-like- receptors [75]. The transition from resting to activated requires rapid upregulation of OXPHOS supported by increased glycolysis. A further role for fatty acid metabolism is evident where disruption of fatty acid synthase (FASN) or ATP citrate lyase (ACLY) results in impaired DC activation, implicating citrate-derived acetyl CoA as key in DC activation [76]. The high metabolic demands of DC are difficult to meet in the TME [74], and DC activation is further suppressed by high lactate levels [77].

## 7. Lymphocytes

Successful antitumor immunity depends on the recruitment and activation of cytotoxic T cells. Non-activated naïve CD8^+^ T cells have modest metabolic demands and principally use OXPHOS to generate ATP [8]. Upon activation, cytotoxic T lymphocytes (CTLs) upregulate glucose, glutamine, and lipid metabolism to support rapid proliferation and effector function [8]. In contrast, memory CD8^+^ T cells upregulate glycolysis to support both lipogenesis and FAO via OXPHOS [78], resulting in increased spare respiratory capacity [79].

High tumor CD4^+^ T cell levels predict adverse tumor outcomes, particularly when coincident with low CD8^+^ T cell levels [80,81,82]. While often treated as a monotypic cell type, significant diversity of function and pathology exists for CD4^+^ T cells in a tumor, with numerous subsets described to predict various disease outcomes [83,84,85]. Similar to CD8^+^ T cells, CD4^+^ T cells adapt their metabolism in line with the stressors and stimuli of the TME [9]. For example, FAO plays a critical role in enabling the function of Treg subsets [16]. A particular role for fatty acid-binding proteins 4 and 5 (Fabp4 and Fabp5) in tissue-resident memory (Trm) T cells is evident in the suppression of Trm expansion in mice lacking both Fabp4 and Fabp5 following viral exposure [86].

Mitochondrial function is also a critical determinant of T cell metabolism, not only as a hub of central carbon, fatty acid, and glutamine metabolism, but also through retrograde signaling via reactive oxygen species (ROS) [87,88,89,90]. Activation of HIF-1α as a result of reduced O_2_ levels, signal transducer and activator of transcription 3 (STAT3) signaling, or TCR signaling plays critical roles in regulating T cell development, proliferation, and function [91]. Aberrant hypoxia signaling in tumors has been shown to impair T cell immunosurveillance via mitochondrial dysfunction [92].

B lymphocytes also play important roles in the development of effective antitumor immunity, with disruption of these processes capable of promoting potent immunosuppressive phenotypes [93,94]. B cell function is intimately tied to metabolic function with discrete metabolic programs supporting development, activation, antibody production, and persistence as memory B cells [95,96]. The formation of tertiary lymphoid structures within the TME is an emerging marker of ICI response in renal cell carcinoma and melanoma [97,98]. Similarly, B cell populations predicted ICI response in sarcoma [99]. Thus, metabolic inadequacy in B cells may suppress ICI response via suppression of B cell function.

## 8. T Cell Activation and Metabolism

### 8.1. T Cell Activation

Naïve T cells are activated following co-ligation of the T cell receptor (TCR) complex, CD4 or CD8 coreceptors, and antigen-loaded MHC [100]. Antigen recognition is accomplished via TCR α and ß chains, whose variable immunoglobulin-like structure allows for specific antigen binding [101]. TCR signal transduction involves tyrosine phosphorylation of the cytoplasmic tails of CD3γ, CD3δ, and CD3ε, and CD3ζ, termed immunoreceptor tyrosine-based activation motifs (ITAMs) [100]. TCR αß ligation promotes conformational shifts in CD3ε and CD3ζ to enhance ITAM availability for phosphorylation by Src family kinases [100]. Lymphocyte-specific protein tyrosine kinase (Lck) activity is regulated by the phosphorylation of two tyrosine residues (Y505 inhibits, and Y394 activates), with a proportion of Lck being constitutively active [102]. The rate of TCR activation by Lck is driven largely by localization of Lck to the immune synapse [102], and CD4/8 coreceptors promote Lck recruitment by direct association [102]. Once localized to the TCR, Lck directly binds ITAM domains, promoting its retention in the immune synapse [100]. Recruitment of Lck to TCR further promotes Lck activation by the exclusion of inhibitory phosphatases, e.g., CD45, either sterically or by promoting membrane detachment [102]. ITAM phosphorylation creates a critical docking site for the ζ-associated protein of 70 kDa (ZAP70), which then phosphorylates linker for activation of T cells (LAT) on tyrosine residues to recruit phosphoinositide-specific phospholipase C gamma 1 (PLCγ1) [102,103]. PLCγ1 then hydrolyzes phosphatidylinositol 4,5-bisphosphate (PIP2) to inositol trisphosphate (IP3) and diacylglycerol (DAG), critical secondary messengers for T cell activation [103]. DAG promotes the activation of extracellular signal-regulated kinase (ERK) and protein kinase C theta (PKCθ) [104], while IP3 promotes Ca^2+^ release [105]. The net result is activation of critical transcriptional responses mediated by nuclear factor of activated T-cells (NFAT) [106], NFκB [107], and Notch1 [108].

**Figure 1:**
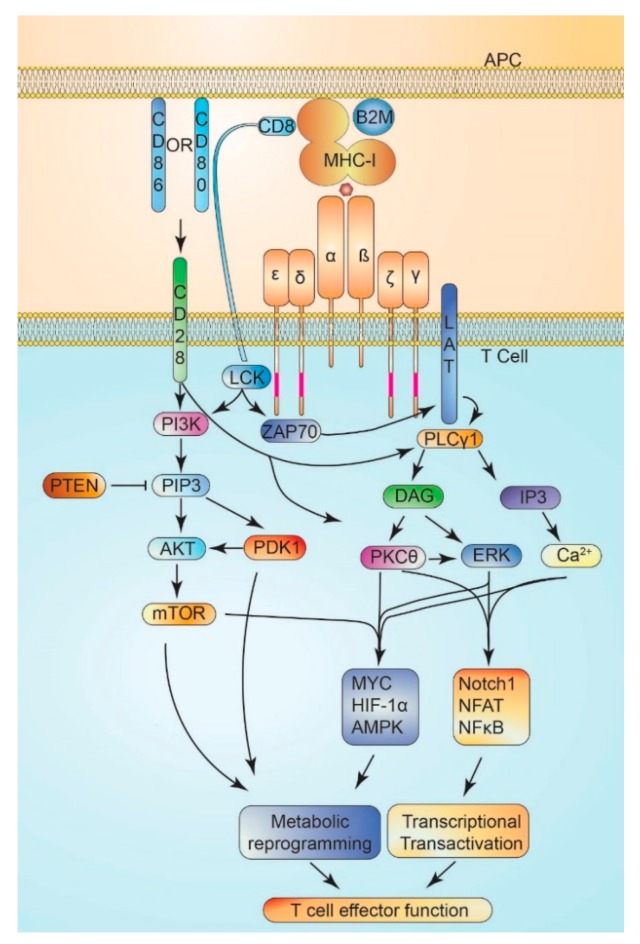
T cell activation and metabolic reprogramming. T cell receptor (TCR) activation occurs following engagement of the αß dimer with peptides presented by MHC-I (or MHC-II)(major histocompatibility complex class I-II), which induces exposure of cytoplasmic domains of CD3ε and CD3ζ. Co-stimulation by CD28 is accomplished by binding of CD80 or CD86 to CD28, activating potent PI3K- mTOR (phosphoinositide-3 kinase-mammalian target of rapamycin) signaling, and reinforcing TCR-mediated signaling. Lck (LCK Proto-Oncogene, Src Family Tyrosine Kinase), tethered by CD8 (or CD4), phosphorylates exposed ITAM (immunoreceptor tyrosine-based activation motif) domains (pink) of CD3γ, CD3δ, CD3ε, and CD3ζ. ZAP70 (zeta-chain-associated protein kinase 70) is recruited to phosphorylated ITAM sites and phosphorylates LAT (linker for activation of T cells). Phospho-LAT recruits PLCγ1 (phospholipase C γ1), which generates the second messengers—DAG (diacylglyercol) and IP3 (inositol triphosphate) from PIP2 (phosphoinsitol bisphosphate), in turn activating ERK (extracellular signal-regulated kinase), PKCθ (protein kinase Cθ), and Ca^2+^ signaling, which promotes transcriptional transactivation by Notch1, NFAT (Nuclear factor of activated T-cells), and NFκB (nuclear factor kappa-light-chain-enhancer of activated B cells) of transcripts required for T cell activation. Simultaneously, multiple pathways converge on the activation of mTOR, HIF-1α (hypoxia inducible factor 1α), MYC (MYC proto-oncogene, bHLH transcription factor), and AMPK (adenosine monophosphate activated protein kinase) signaling to promote metabolic reprogramming required by T cells for effector function.

Additionally, transcription factors, such as T-bet (T-Box Transcription Factor 21) [109], IRF4 (Interferon Regulatory Factor 4) [110], and FOXP3 (forkhead box P3) [111], have been shown to regulate T cell metabolic shifts. In addition to activation of the TCR, co-stimulation is required for the full activation of T cells, most potently achieved by binding of CD28 with CD80 or CD86 [103]. CD28 activation promotes the activation of PI3K-AKT-mTOR signaling [103], linking cellular metabolism to T cell activation. Indeed, CD28 costimulation enhances glucose consumption and mitochondrial priming to supply acetyl CoA to enable rapid expansion [112,113]. As T cells adapt to meet the metabolic demands of activation, proliferation, and effector function, they increase glucose and glutamine consumption, with increased OXPHOS and fatty acid metabolism [114]. Indeed, electron transport chain activity has been proposed as a metabolic checkpoint for T cell activation [115]. In summary, mediators of such metabolic reprogramming are essential for T cell activation, including mTOR, HIF-1α, AMPK, and MYC, highlighting the necessity of T cell metabolic reprogramming to effector function [114] (Figure 1).

### 8.2. Costimulatory Signaling

In addition to CD28, other costimulation can occur through related signaling pathways to induce T cell activation and associated metabolic reprogramming.

OX40 (CD134) is expressed primarily on activated CD4^+^ T cells, and CD8^+^ T cells to a lesser extent [116]. OX40 binds to OX40L, which is expressed on various APCs, including DCs and B cells [117]. When engaged by OX40L, OX40 activates PI3K, NFκB, and Ca^2+^ signaling to promote T cell proliferation and inhibit Treg formation [117]. OX40-stimulatory antibody therapy has shown efficacy in combination with other cancer immunotherapy, with several ongoing clinical trials [118].

4-1BB (CD137) is basally detectable in most cell types and strongly inducible in DCs and Tregs [119]. Engagement with 4-1BBL induces robust NF-κB activation to promote CTL activation [120]. 4-1BB costimulation also promotes increased fatty acid and glucose consumption while promoting mitochondrial biogenesis [121,122]. Indeed 4-1BB co-stimulation has been shown to support antitumor immunity by promoting T cell mitochondrial function [122]. Immunotherapy using 4-1BB agonist antibodies has long been studied [123] with several ongoing clinical trials [124].

CD40 is widely expressed, including on APCs, such as DCs [125]. CD40 binds to CD40L (CD154), which is expressed on activated T-cells [125]. CD40 engagement with CD40L upregulates cytokine production in DCs and enhances antigen presentation, licensing them to mature [125]. Dual costimulation of 4-1BB and OX40 enhanced glucose uptake, glycolysis, and OXPHOS [126]. In B cells, CD40 signaling stimulates germinal center formation, promotes immunoglobulin isotype switching, enhances antigen affinity, and promotes memory B cell formation [127]. Anti-CD40 therapy inhibits tumor growth and induces APC maturation to promote antitumor response, with several ongoing clinical trials [128]. CD40 agonist antibodies may direct tumor cell opsonization and antibody-dependent cellular cytotoxicity (ADCC) to further promote antitumor effects [125].

Interleukin-2 (IL-2) potently induces T cell survival and cytotoxicity via engagement of its receptors and induction of PI3K, STAT1, -3, and -5, and ERK signaling [129]. High dose IL-2 has been approved as monotherapy for some metastatic disease, and multiple clinical trials are underway to investigate IL-2 in combination with chemotherapies or other immunotherapies [129].

Inducible T-cell COStimulator ICOS, CD278) is induced following T cell activation and binds ICOSL expressed on APCs and non-immune cells, such as endothelial cells [130]. Ligand engagement upregulates T cell proliferation and cytokine secretion via activation of PI3K and mitogen-activated protein kinase (MAPK) signaling [130]. ICOS costimulation promotes robust activation of mTOR signaling in follicular T cells [131,132]. ICOS signaling is implicated in the generation of suppressive Treg populations [133,134]. Hence, both antagonistic and agonistic ICOS immunotherapies have been developed and are currently in clinical trials [130].

GITR (glucocorticoid-induced tumor necrosis factor receptor) is expressed at high levels following activation in both CD4^+^ and CD8^+^ subsets [135]. GITR signaling is induced following binding of GITRL, found on APCs and endothelial cells, and results in the activation of NFκB and MAPK signaling. GITR agonists have been shown to potently suppress Treg function while activating CD4^+^ and CD8^+^ effector T cells [135]. While limited success has been seen with GITR agonist monotherapy, it may prove useful in combination with other immunotherapies [136].

ICIs attempt to promote T cell activation and thus drive metabolic reprogramming in T cells to enable effective antitumor immune response. Conceptually, metabolic therapies that enable such reprogramming are essentially also immunotherapies, and may, therefore, be synergistic with ICIs.

## 9. Immune Checkpoint Signaling

Immune checkpoints refer to numerous inhibitory pathways found in immune populations that are critical to self-tolerance and modulation of the immune response [137]. Several immune checkpoints have been found to involve suppression of PI3K-AKT-mTOR signaling or metabolic responses of T cells following co-stimulation. ICIs attempt to promote T cell activation and thus drive metabolic reprogramming in T cells to enable effective antitumor immune response. Conceptually, metabolic therapies that enable such reprogramming are essentially also immunotherapies, and may, therefore, be synergistic with ICIs.Among the most prominent therapeutic targets for immune checkpoint blockade are CTLA-4 and PD1. CTLA-4, expressed exclusively on T cells, is a co-inhibitory receptor that is induced following T cell activation [138,139]. CTLA-4 has a higher binding affinity for CD80 and CD86 than does CD28, leading to blocked T cell activation via competition for co-stimulatory signals [140].

PD1, also expressed on B and myeloid cells, is induced on T cells by antigen stimulation [141]. The PD1 C-terminal contains an immune receptor tyrosine-based inhibitory motif (ITIM) and an immune receptor inhibitory tyrosine-based switch motif (ITSM) [139,142]. PD-L1-bound PD1 recruits SHP1 (Src homology 2 domain-containing protein tyrosine phosphatase 1) to its ITIM and ITSM domains and SHP2 to the ITSM domain, thus attenuating both CD28-mediated PI3K signaling and TCR-mediated ZAP70 signaling to suppress T cell activation [143]. PD1 signaling suppresses PI3K-AKT-mTOR signaling to impair glucose and glutamine utilization in T cells [143]. Indeed, PD1 signaling in T cells promotes FAO via the upregulation of AMPK signaling [144], with significant mitochondrial dysfunction and impaired glycolysis in PD1^+^ TILs capacity [92]. This may be STAT3 mediated in part [145]. Further, PD1-mediated metabolic insufficiency has been demonstrated to mediate T cell exhaustion [146]. PD-L1 and B7-H3 promote glycolysis in cancer cells via PI3K/AKT/mTOR and HIF-1α signaling, respectively [147,148]. Thus, metabolic remodeling is fundamental to immune checkpoint function and to the efficacy of immune checkpoint inhibition. In addition to cell surface expression, exosomal PD-L1 may have important roles in promoting tumor immune evasion [149]. Numerous clinical trials are underway combining drugs intended to promote metabolic reprogramming with immune checkpoint inhibition, as summarized by Li et al. [150]. High levels of PD-L1 expression in some lymphomas may promote a higher response rate to ICI therapy (80–90%) than is seen in solid tumors [2]. An intriguing role for PD1 signaling in myeloid cells has recently been uncovered, wherein PD1 signaling in tumor-bearing mice appears to drive the metabolic suppression of granulocytic bone marrow precursors and promotes the generation of myeloid-derived suppressor cell (MDSC) populations [151]. Antitumor immunity following the deletion of PD1 in myeloid cells alone is as effective as the whole animal knock out [151]. 

B7-H3 is another member of the B7/CD28 family frequently observed on tumor cells and has been implicated in both costimulatory and coinhibitory signaling in T cells [152,153]. The mechanism of action of B7-H3 remains incompletely understood, with no known receptor identified as responsible for T cell responses [154]. B7-H3 expression suppresses cytotoxic T cell function in ovarian cancers insensitive to blockade of PD1 [155]. Anti-B7-H3 therapies have entered clinical trials in a number of cancer types (NCT02628535, NCT02923180, and NCT01391143).

Lymphocyte activation gene-3 (LAG-3) and T cell immunoglobulin-3 (TIM-3) are expressed on CD4^+^ and CD8^+^ T cells as well as natural killer cells [156]. LAG-3 is highly related to CD4 and shares its CD3 association and MHC-II binding; however, it lacks the ability to recruit Lck [157]. The signal transduction leading to reduced T cell cytokine production and proliferation by LAG-3 is not fully understood, but there may be a role for T cell cytosolic signals being transduced through LAG-3 to suppress DC function via MHC-II binding and ERK signaling, resulting in reduced cytokine production and loss of CD86 expression [158]. LAG-3 targeted therapies are currently involved in several clinical trials [159].

TIM-3 has several described ligands, including galectin-9 (Gal9), binding of which results in phosphorylation of two cytosolic tyrosine residues (Y256 and Y263) [160]. These residues are required for human leukocyte antigen B (HLA-B)-associated transcript 3 (BAT3)-mediated recruitment of Lck to TIM-3, which, in turn, supports TCR activation [160]. Phosphorylation of these residues following ligand binding results in loss of Lck and suppression of TCR activation [160,161].

A2A receptor (A2AR) activation downregulates the immune response in response to adenosine, an endogenous nucleoside found in inflammatory environments and around tumors [162]. ATP in the TME is readily converted to adenosine by CD39 and CD73, among other ectonucleases, which can then modulate a range of immune cell populations in part via activation of A2AR [163]. Following activation via extracellular adenosine, A2AR-mediated accumulation of intracellular cAMP is followed by the suppressed function of natural killer [164,165] and T cells [166]. Significant preclinical support for targeting A2AR, CD39, or CD73 in cancer has prompted several clinical trials [166].

**Figure 2:**
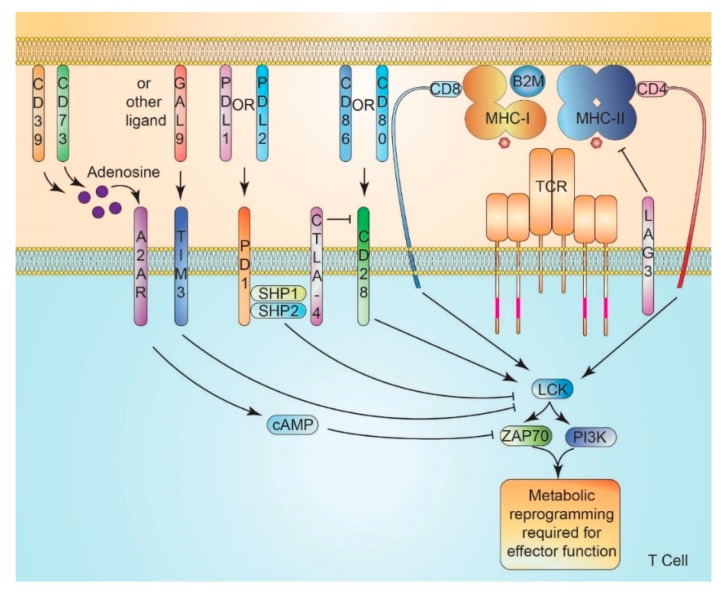
Immune checkpoint signaling abrogation of T cell receptor signaling and metabolic adaptation. Competition between CTLA-4 (cytotoxic T-lymphocyte-associated protein 4) and CD28 for CD80 and CD86 results in loss of CD28 costimulatory signals. Binding of PD-L1 (programmed death ligand 1) or PD-L2 (programmed death ligand 2) to PD1 (programmed death-1) recruits SHP1 and SHP2 (Src homology region 2 (SH2)-containing protein tyrosine phosphatase 1/2) to suppress TCR activation of Lck (LCK Proto-Oncogene, Src Family Tyrosine Kinase). Binding of GAL9 (galectin 9)(or other ligands) to TIM-3 (T cell immunoglobulin and mucin domain-containing protein 3) results in the detachment of BAT3 (HLA-B-associated transcript 3) and loss of Lck localization to the immune synapse. LAG3 (Lymphocyte Activating 3) antagonizes antigen presenting cell function via suppressive interaction with MHC-II (major histocompatibility complex class II), with additional yet poorly understood T cell-intrinsic signaling likely. The production of adenosine from extracellular ATP by CD73 and CD39 promotes the activation of A2AR (adenosine A2A receptor), which suppresses T cell activation via cAMP (cyclic AMP) production.

IDO metabolism of tryptophan represents another targetable immune checkpoint due to its role within the kynurenine degradation pathway in DCs, monocytes, and macrophages [167]. IDO expression by innate immune cells suppresses T-cell survival and proliferation via tryptophan starvation and kynurenine-induced cell signaling [168]. IDO inhibitors have seen success clinically, usually in combination with anti-CTLA-4 or anti-PD1 antibodies [169] (Figure 2).

## 10. Metabolically-Mediated TME Immunomodulation

### 10.1. Lactate

High concentrations of lactate, an end product of glycolysis, are critical regulators of the TME [170]. Tumor masses are major contributors to high lactate concentrations, producing up to 40 times more lactate than healthy tissues [171]. High lactate levels and reduced pH contribute to immunosuppression within the TME and may play a role in the limited efficacy of immunotherapies [170,172,173]. Indeed, lactate production correlates with reduced immunosurveillance, limited immunotherapy response, and poorer clinical outcomes [174,175,176].

The export of lactate results in the acidification of the TME [177]. Lowering in vitro conditions to pH levels similar to tumor masses establishes an anergic state in human and mouse-specific CD8^+^ T lymphocytes [178]. Recent work has identified V-domain Immunoglobulin suppressor of T cell activation (VISTA), an inhibitory immune checkpoint molecule, to suppress T cell activity in response to reduced pH [179]. While prolonged incubation in high concentrations of lactic acid leads to cell death of up to 60% of CTLs, the treatment of CTLs in an acidic environment devoid of lactate has no such effect, suggesting TME acidification alone is insufficient for lactate-mediated immunosuppressive effects [180]. Similarly, studies in culture have shown that high lactic acid suppresses T lymphocyte function [175,180]. APCs necessary for the activation of CTLs are also affected by high lactate concentrations. Antigen presentation by DCs is inhibited by lactic acid [181], providing evidence for T cell-extrinsic suppression of immunosurveillance. Tumor-derived lactic acid also promoted in vitro polarization of TAMs towards their M2-like phenotype [67,182]. Lactic acid pre-treated macrophages inhibit CD8^+^ T-cell proliferation and induce expression of arginase, which independently impairs T-cell response [67,183].

**Figure 3:**
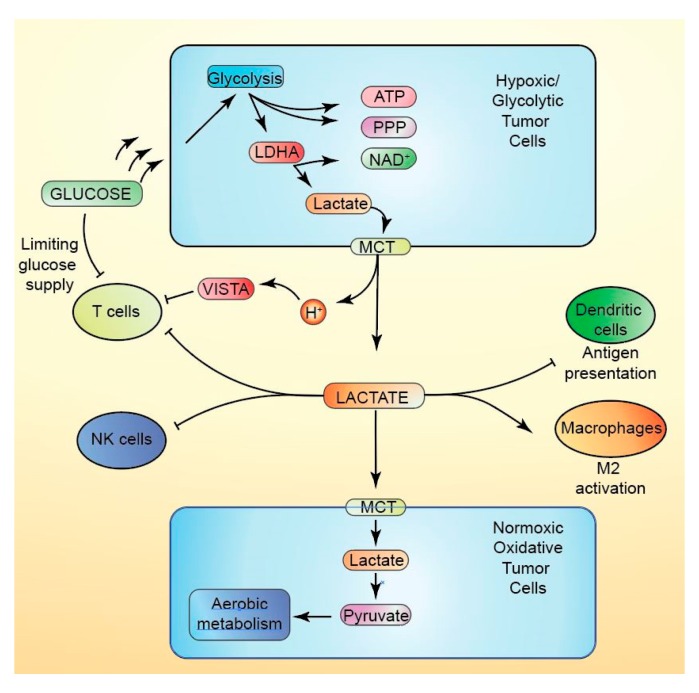
Lactate metabolism in the tumor microenvironment. Rapid glucose uptake by tumor cells, particularly hypoxic cells, depletes glucose from the tumor microenvironmen (TME), thus limiting glucose availability for T cells. Rapid glycolysis in tumors provides a ready supply of ATP and anabolic intermediates; NAD^+^ is regenerated to avoid reductive stress by the production of lactate by LDHA (lactate dehydrogenase A). Lactate is then exported by MCT (monocarboxylate transporter) family members to the TME, where it accumulates and acidifies the TME. Oxidative tumor cells can take up lactate and convert it to pyruvate to supply acetyl CoA for aerobic metabolism. High levels of lactate directly suppress T cell and natural killer cell function in the TME. The acidification of the TME suppresses T cell response via activation of VISTA (V-domain Immunoglobulin suppressor of T cell activation) signaling. Lactate further suppresses TME immune surveillance by blocking antigen presentation by dendritic cells and promoting M2 activation of macrophages.

The enhanced expression of lactate dehydrogenase A (LDHA) is associated with numerous cancer types and is critical for lactate efflux [184]. Reducing expression of LDHA in melanoma cells leads to impaired growth of tumors in immune-competent C57BL/6 mice, yet has little effect in immunodeficient mice, suggesting that lactate production greatly suppresses the host’s immune response [175]. Inhibition of lactate production and pH buffering restores T-cell function, reduces Treg activation markers, and increases the efficacy of immunotherapy [178,185,186]. These findings suggest that lactate production and the subsequent accumulation of lactic acid within the TME impede effective immune response. Understanding the mechanisms through which lactate may mediate these effects will continue to shed light on the emerging role of the TME and lactate metabolism in immunotherapy resistance (Figure 3).

### 10.2. Hypoxia

Highly proliferative cells require substantial oxygen and nutrient supply [187]. However, the development of tumor masses often occurs at a faster rate than the vasculature, resulting in an environment deficient in oxygen—i.e., hypoxia [6,188]. Hypoxia in the TME has been implicated in tumor cell invasion, metastasis, immunosuppression, and therapeutic resistance [189,190,191]. HIF-1α is a transcriptional master regulator of the cellular hypoxic response [192]. Canonical HIF-1 target genes include glucose transporters, glycolytic enzymes, and vascular endothelial growth factor (VEGF) [192]. Hypoxia and the subsequent stabilization of HIF-1α promote phagocytotic and inflammatory functions of the innate immune system [193,194,195]. However, hypoxia in the TME also results in innate immune-driven immunosuppression via TAM polarization and suppression of adaptive immune response [196,197,198]. Furthermore, hypoxia promotes PD-L1^+^ tumor-associated MDSCs, resulting in T cell suppression, which is mitigated by PD-L1 blockade [199]. HIF-1α also increases both the number and suppressive properties of Tregs via the upregulation of FOXP3 [200,201]. Additionally, CD4^+^ and CD8^+^ effector T cell function is impaired under hypoxic conditions, in part due to metabolic suppression [189,202,203]. These findings suggest that hypoxia in the TME drives immunosuppression and that therapies that relieve such suppression, either by promoting T cell metabolic function or by promoting immune independent death of hypoxic tumor cells, may synergize with ICIs.

### 10.3. Arachidonate Metabolism

A wide range of cells in the TME generate inflammatory lipid mediators via arachidonic acid/arachidonate metabolism [204]. Arachidonate is released from membrane phospholipids by members of the phospholipase A2 (PLA2) superfamily of enzymes and subsequently metabolized by lipoxygenases or cyclooxygenases. Lipoxygenases yield leukotrienes and lipoxins, while cyclooxygenase 1/2 metabolism produces prostaglandins (PG; e.g., PGD2, PGE2, prostacyclin) and thromboxanes (e.g., thromboxane A2, PGF2α) [205]. While the complex regulation of PG production in the TME is beyond the scope of this review (the reader is referred to Kobayashi et al. [204]), PG signaling has been shown to promote adverse tumor outcomes via immune suppression, particularly PGE2 [206,207]. Moreover, blockade of COX activity with nonsteroidal anti-inflammatory drugs (NSAIDs) reduces the rates of metastasis in breast and colon cancers [208], while the combination of NSAIDs with ICI therapy is also supported by promising preclinical data [206].

### 10.4. Short-Chain Fatty Acid Metabolism

Energy regulation, immune function, and ICI therapy outcomes have all been associated with fecal microbiome composition [209]. For example, fecal microbiome composition varied significantly between responder and nonresponder melanoma patients receiving ICI therapy [210]. Additionally, the modulation of gut or intratumoral microbiomes alters the antitumor immune response [211,212]. Indeed, an intact gut microbiome appears to be crucial for ICI response [213,214], while antibiotic use reduced the efficacy of ICI in both mouse [213] and human studies [215]. The microbiome produces many important metabolites, including short-chain fatty acids (SCFAs). SCFAs have been reported to modulate critical immune and endothelial cell functions via the activation of G protein coupled receptor signaling, inhibition of histone deacetylase activity, and supply of anabolic substrates [216,217]. It is clear that SCFA production by intestinal microbes regulates immune function; however, to date, little work has been conducted to determine whether microbial SCFA production directly influences the likelihood, amplitude, or duration of ICI response.

## 11. Tumor Cell-Intrinsic Immunotherapy Resistance

### 11.1. Metabolically-Mediated Immune Evasion

**Figure 4:**
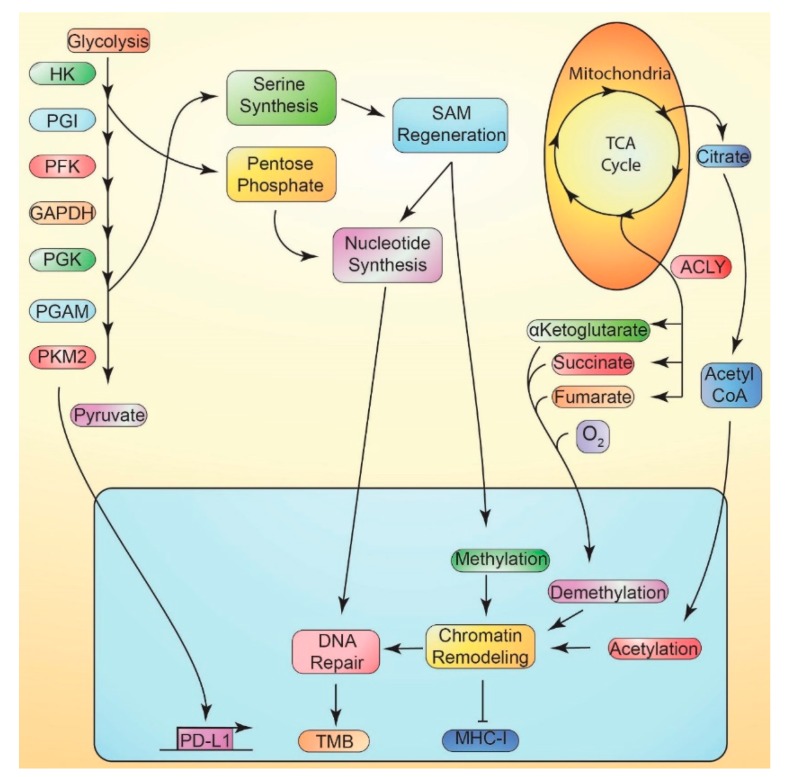
Tumor cell-intrinsic metabolic regulation of immune escape. High rates of glycolysis in tumor cells, enabled by increased expression of PKM2 (pyryvate kinase muscle 2), supply intermediates required for serine biosynthesis and pentose phosphate metabolism. PKM2 can directly promote the expression of PD-L1 (programmed death ligand 1) independent of its regulation of glycolytic metabolism. Pentose phosphate metabolism (PPP) is required for nucleotide biosynthesis. Serine biosynthesis readily supplies carbon for the regeneration of S-adenosyl methionine (SAM), which is required for both nucleotide biosynthesis and chromatin remodeling via methylation. Nucleotide biosynthesis supplies required nucleotides for DNA repair following damage. Tricarboxylic acid cycle metabolic intermediates also regulate chromatin remodeling. Citrate, exported to the cytoplasm, supplies substantial amounts of acetyl CoA via ATP citrate lyase (ACLY), required for acetylation. Alpha-ketoglutarate, succinate, fumarate, and oxygen regulate a range of demethylation reactions, either as substrates or as inhibitors. Chromatin remodeling is essential to efficient DNA repair, failure of which drives an increase in TMB. Further, MHC-I (major histocompatibility complex class I ) is epigenetically suppressed by many cancer cells.

An effective response to immunotherapy requires that tumor immunoediting is overcome. Several cancer cell-intrinsic metabolic processes direct immune evasion, and thus potentially resistant to immunotherapy. Cancer cells commonly upregulate the alternative splicing of pyruvate kinase muscle (PKM2) from PKM pre-mRNA to enable further aerobic glycolysis [218]. The catalytic activity of PKM2 is regulated by its oligomerization states, with significantly higher activity as a tetramer than as a dimer. Thus, regulation of PKM2 oligomerization allows fine control of glycolytic metabolism [219]. PKM2 has been shown to promote the expression of PD-L1 in cancer cells, macrophages, and DCs [220,221] and to recruit MDSCs to tumors [222]. Though there is limited understanding of the impact of such compounds on immunotherapy response, drugs that disrupt the dynamic regulation of PKM2 oligomerization, e.g., by promoting tetramerization, have been shown to impair tumor growth [223] (Figure 4). Recent work has shown tumor cell sphingosine kinase-1 to be an important mediator of resistance to both PD1 and CTLA-4 ICI in mice [224].

### 11.2. Suppression of MHC-I vs. Metabolism

MHC-I expression is critical for effective tumor immunosurveillance and is frequently suppressed in cancer. MHC-I antigen presentation is controlled by epigenetic methylation and acetylation [225,226,227], both highly metabolically-dependent processes [228]. Tumor cell aerobic glycolysis has also been associated with the reduction of MHC-I expression [229]. Induction of tumor cell metabolic stress, through limiting either oxygen or glucose, has reduced expression of MHC-I and dampened interferon γ induction of MHC-I in melanoma and lung cancer cell lines [230]. In leukemia cell lines, suppression of glycolysis or enhanced OXPHOS has promoted the expression of MHC-I [231]. Metformin treatment has promoted MHC-I expression via mitochondrial biogenesis in breast cancers, but not in nontransformed breast epithelial cells [232]. The potential synergy between metformin and immunotherapies is currently under investigation in a number of clinical trials [150]. Thus, metabolism may play a key role in the regulation of MHC-I antigen presentation and immunotherapy response in tumors (Figure 4).

### 11.3. Tumor Mutational Burden

Cellular metabolism is fundamentally linked to DNA repair through chromatin remodeling, biosynthetic production of nucleotides, and regulation of redox status [233]. Genomic instability, i.e., an increased rate of mutation and impaired mutational repair, has long been recognized as a hallmark of cancer [25,234]. Tumor metabolism drives chromatin remodeling and epigenetic modifications in several ways, including by supplying acetyl and methyl groups, maintaining NAD^+^/NADH levels, and regulating several metabolites which act as cofactors or inhibitors of key enzymes, such as α-ketoglutarate, succinate, fumarate, and 2-hydroxyglutarate [235]. Importantly, a higher tumor mutational burden (TMB) may increase the likelihood of expression of immunogenic neoantigens by tumor cells, thereby inducing a more robust response to immunotherapy. Thus, TMB is rapidly emerging as a biomarker of immunotherapy response, with higher TMB associated with improved response to checkpoint inhibition in several clinical trials [236]. Hence, tumor metabolism may influence resistance to checkpoint inhibition by the modulation of TMB (Figure 4).

## 12. Interactions between Systemic Metabolic Perturbations and Intrinsic Immunometabolism

### 12.1. Obesity

Metabolic status is crucial to the efficacy of tumor immune surveillance and immunotherapy. Obesity is a complex disease characterized by excessive adipose tissue [237] and is a significant risk factor for numerous cancer types [238]. In addition to storing triglycerides, white adipose tissue (WAT) acts as an endocrine organ [239]. Dysfunctional adipokine signaling is a major contributor to obesity-linked metabolic syndrome [240,241]. Adipokine profiles of obese patients typically include lower levels of adiponectin, higher levels of leptin, and increased proinflammatory cytokines (e.g., IL-6 and tumor necrosis factor α (TNF-α)) [242,243]. Adiponectin is the only adipokine negatively correlated with fat mass and has been shown to improve insulin sensitivity by upregulating AMPK [244]. Thus, inflammation is further aggravated in obesity by a decrease in adiponectin, which is associated with insulin-sensitizing, anti-inflammatory, and anti-apoptotic effects [245]. Leptin is a peptide hormone secreted by WAT, skeletal muscle, gastric cells, and enterocytes [246]. Often referred to as the ‘satiety hormone’, this regulatory hormone inhibits hunger by acting on the leptin receptors in the hypothalamus [247]. Despite leptin’s long-term effect of limiting food intake, a paradoxical hallmark of obesity is hyperleptinemia [248]. This association is often cited as evidence of leptin resistance; however, the relationship between obesity and leptin resistance remains widely disputed [249]. Aberrant adipokine signaling in obesity is associated with chronic pro-inflammatory signaling that promotes the impairment of immune function [250].

It has been extensively demonstrated that obesity drives chronic low-grade systemic inflammation [251]. In addition to adipose tissue, T-cells, B-cells, and macrophages are important contributors to the production of obesity-driven inflammatory cytokines [252,253,254]. Among these cytokines, IL-6, TNF-α, and IL-1β function in the recruitment of T cells, dendritic cells, and macrophages to adipose tissue [255]. The production of TNF-α and IL-6 by WAT macrophages is strongly linked to the obesity-driven chronic inflammatory state [255]. Interestingly, obesity also promotes the recruitment of myeloid-derived suppressor cells to both adipose and tumor tissue [256].

Importantly, immune cell activity is modulated by obesity-induced inflammation, resulting in dramatically different response profiles to immunotherapies in preclinical models [257]. Both obese mice (induced by diet) and ob/ob mice exhibit strikingly higher toxicity following administration of a CD40 agonist in combination with IL-2 than has been observed in lean mice [258]. Obese mice treated with anti-PD1 have shown greater tumor response compared to lean controls, potentially due to leptin-induced expression of PD1 [259,260,261,262].

Much work has shown that leptin can promote the activation of T cells [263] while also promoting MDSC formation in obesity [264]. Pathological levels of leptin induced by obesity have been identified as limiting ICI response, while leptin receptor blockade has improved ICI response [265]. Viral-mediated leptin overexpression in tumor cells promotes T cell activation and antitumor immunity, which has exceeded that achieved by oncolytic virus alone [266]. Reduction of leptin during fasting promotes T cell dysfunction and metabolic reprogramming, which is rescued by exogenous leptin [267]. Intermittent fasting produces alternating cycles of high and low leptin, thus supporting the anticancer effects of calorie restriction (CR) while providing sufficient leptin to promote immune cell function [267]. Conversely, high levels of leptin in obese mice have been shown to promote breast cancer growth via suppression of CTL glycolysis and upregulation of FAO [145]. Despite obesity-driven inflammation and its potential for immunosuppression, immunotherapy survival rates are higher with obesity [259,268,269]. Retrospective analysis of clinical data indicates that in some cancer types, obesity is associated with improved survival following immunotherapy, but not following other therapies [260,261]. Indeed, there is a 52% increase in overall survival in obese renal cell carcinoma patients with PD-L1 positive tumors compared to normal-weight patients, while, in PD-L1 negative tumors, this difference is negligible [270]. Similarly, overweight/obese patients with multiple cancer types treated with ICIs have exhibited greater objective response rate, time to treatment failure, progression-free survival, and overall survival, yet have been more than twice as likely as normal-weight patients to experience an immune-related adverse event [260,261]. Together this data indicates that while chronic inflammation/immunosuppression in obesity may promote tumor growth, it may also increase susceptibility to ICI therapy in select populations.

### 12.2. Caloric Deficit

Like obesity, caloric restriction (CR) is a potent modulus of immune function; while obesity promotes increased inflammation and T cell exhaustion, CR reduces chronic inflammation and improves the function of immune cells. Thus, while both CR and obesity appear to improve ICI response, the mechanisms through which they act are likely only partially overlapping. Moderate CR has been long understood to have potent anticancer effects [271,272], with recent developments pointing to important roles for CR in aging and immune function [273]. CR partially reverses immune dysfunction induced by obesity [274,275,276], with considerable improvements in immunosurveillance reported [277]. CR pleomorphic immune effects, with both protective and deleterious effects reported. CR in aged mice has enhanced the maintenance of hematopoietic stem cell populations and impaired the differentiation of some lymphocyte populations, in part mediated via suppression of insulin like growth factor 1 and IL-6, respectively [278]. Conversely, in non-human primates, CR has protected against the development of T cell senescence [279]. Indeed, the dampening of mTOR signaling in *Drosophila* following CR enhanced innate immune function [280]. T cell dysfunction with aging has been widely reported and, in mice, is associated with a decline in anti-OX40 immunotherapy response [281]. CR promoted the maintenance of T cell function and anti-OX40 responsive aged CD4^+^ T cell populations [282]. CR in mice has induced signaling between eosinophils, macrophages, and adipose tissue to promote adipose browning required for metabolic improvements, including increased thermogenesis, improved glucose tolerance, and greater fat loss [283]. Tumor-derived IL-6 has been shown to suppress hepatic ketogenesis, promoting glucocorticoid-mediated immune suppression and resistance to immunotherapy in mice with a caloric deficit [284].

Chronic CR in cancer patients is complicated by the often poor nutritional status of patients undergoing therapy [285]. Hence, intermittent CR or fasting approaches for short periods of time, followed by consumption of an otherwise healthy diet, offer an attractive alternative [286]. A critical component of “Beneficial CR” is the continued adequate supply of micronutrients and protein. This is absent from the states of malnutrition, which have been shown to suppress both T cell number and function, likely related to the concomitant increase in infection risk in malnourished patient populations [287]. Similarly, both sarcopenia and cachexia have been associated with poorer clinical response to immunotherapies [288,289,290].

Intermittent fasting mediates a range of anticancer effects, many of which rely on the differential response of tumor cells and normal tissue to fasting. Some preclinical work has indicated that intermittent fasting promotes antitumor immunity, both by reprogramming TAMs [291] and by enhancing CD8^+^ T cell cytotoxicity [292]. Indeed, an intermittent fasting approach has demonstrated substantial benefit when used in combination with chemotherapy and immunotherapy [293].

## 13. Conclusions

ICIs have yielded tremendous successes and revolutionized the field of immuno-oncology. However, a high rate of non-responders remains a considerable limitation, particularly in the treatment of solid tumors. When coupled with the significant toxicity and high cost of ICIs, finding successful approaches to improving response rate and duration to these therapies is a key problem that must be addressed. Herein, we argued that ICI therapy is, in part, a metabolic therapy—one that is greatly limited by the hostile metabolic environment of the TME. Further, we detailed some of the cooperation and competition within the TME and its potential to augment or impair immunotherapy response. As we discussed throughout this review, T cell activation is requisite for ICI to be effective, yet for T cell activation to be effective, reprogramming of T cell metabolism and a large upregulation of nutrient consumption are critical. Finally, we examined obesity/host nutritional status and dietary approaches currently being investigated for their potential to impact tumor immunosurveillance. We posit that understanding how the metabolic interplay within the TME—as well as the impact of ICI on tumor cell and immune cell metabolism—informs immunotherapy response, and ultimately resistance, allowing for the development of novel strategies to improve patient outcomes following immunotherapies.
